# Improving the service quality of cross-border e-commerce: How to understand online consumer reviews from a cultural differences perspective

**DOI:** 10.3389/fpsyg.2023.1137318

**Published:** 2023-07-14

**Authors:** Linlin Han, Xu Han

**Affiliations:** ^1^Faculty of Business Information, Shanghai Business School, Shanghai, China; ^2^School of Information Engineering, Minzu University of China, Beijing, China

**Keywords:** cross-border electronic commerce, cultural differences, online consumer reviews, service quality, empirical analysis

## Abstract

**Introduction:**

Cross-border e-commerce (CBEC) consumers come from different countries; thus, cultural differences may affect their evaluations and perceptions of service quality. This paper follows Hofstede’s framework as a theoretical anchor to explore how to use online consumer reviews that reflect cultural differences to improve the service quality of CBEC.

**Methods:**

First, based on a latent Dirichlet allocation model, 14 service quality issues that consumers are concerned about in CBEC were identified. Second, a generalized ordered logistic regression model was explored to analyze the cultural influences on consumer sentiment orientation. Finally, the effect of each cultural dimension on consumer service quality perception in CBEC was evaluated by employing a binary logistic regression model.

**Results:**

The results showed that consumers paid more attention to the service quality of logistics service, customs efficiency and tariff, shopping experience, and so on. Cultural dimensions significantly impacted consumers’ emotional tendencies. Moreover, cultural dimensions had significant impacts on consumers’ service quality perception (e.g., logistics service, trust in sellers, customs disputes, and cell phone performance). Still, consumers’ quality perceptions of some services (e.g., cell phone functions, items as described, logistics package quality, and gifts) were less affected by cultural dimensions.

**Discussion:**

Our findings not only provide new perspectives for CBEC consumer behavior studies on quality improvement but also provide practical implications for CBEC enterprises.

## 1. Introduction

In recent years, due to the ongoing development of internet technology and the intensifying of economic globalization, cross-border electronic commerce (CBEC) has become a prevalent international trade pattern (Meertens et al., 2020) and an important growing mode of foreign trade (Mou et al., 2020). The B2C electronic market is an important model of CBEC in many countries. In the B2C model, since sellers and buyers come from different countries, the service quality of CBEC is generally affected by logistics efficiency (Agatz et al., 2008), tariff, customs clearance efficiency (Zhao, 2020), communication quality between buyers and sellers, exchange rates risk, return processes, and so on Shi et al., 2020; Wang et al., 2020). CBEC consumers have shifted from buying products to buying bundles of products and related services (Niu et al., 2019). Kotha et al. (2004) claimed that the quality of the online shopping experience—comprising website usability, customer confidence in the web business, the selection of goods, extent of customer confidence, and effectiveness of relationship services—represents a competitive advantage for e-commerce firms. High service quality not only can directly enhance positive behavior intentions, but also can heighten customer satisfaction, and then indirectly strengthen purchase intention and loyalty to electronic retailers (Carlson and O’Cass, 2010; Gounaris et al., 2010). Designing better product-launch strategies to improve service quality is a common concern for CBEC enterprises (Wang et al., 2019).

Service quality can be conceptualized as the difference between perceived service performance and expected service level (Donthu and Yoo, 1998). In the B2C model of CBEC, consumers come from all over the world and possess different cultural traits. Cultural differences may influence their perception of service quality. Leon (2019) found that individuals sharing the same history, socioeconomic, and political environments developed similar value judgments and distinguished them from others. Culture influences consumer behavioral intentions and perception of service quality (Furrer et al., 2000; Ahmad et al., 2021). Consumers with different cultural traits vary in their expectations and perceptions of the overall service quality (Winsted, 1997; Donthu and Yoo, 1998; Furrer et al., 2000). Cultural dimensions are associated with overall service quality expectations and perceptions.

With globalization, cross-cultural business management has attracted widespread attention. Because of cultural differences, some international marketing strategies may be successful in some nations but unsuccessful in others (Jin et al., 2008; Ladhari, 2008; Lu et al., 2017). The growth of the internet has increased the need for cross-cultural research even more, as business success depends on global consumers and effective online marketing strategies (Alcántara-Pilar et al., 2018; Cheng et al., 2019). To improve the quality of services, businesses must comprehend how services affect consumer behavior from the standpoint of cultural differences (King et al., 2014) and develop individualized operation methods around cultural differences. Furthermore, CBEC enterprises need to analyze consumers’ behavioral intentions and provide culturally adapted services (Sinkovics et al., 2007) to improve the satisfaction of customers from different cultures.

The majority of prior studies that have examined CBEC consumer behavior have used the questionnaire survey method (Huang and Chang, 2019). With the rapid development of the internet and Web 2.0 applications, many e-commerce platforms provide both online consumer reviews and professional reviews for products to promote sales and improve the quality of products or services (Zhou and Duan, 2016). Online consumer reviews are opinions of consumers on products or services (Zhang and Lin, 2018; Lee et al., 2021; Yi and Oh, 2022). Professional reviews, on the other hand, are generated by professional experts (Zhou and Duan, 2016; Perano et al., 2021). Consumer reviews are given more weight by consumers when making purchase decisions than expert reviews (Tsao, 2014), and they also more accurately reflect the caliber of products and services (Zhang et al., 2010). Online consumer reviews are important information resources for CBEC enterprises. Consumers write reviews on their initiative after shopping for products or services; thus, reviews represent how actual experiences consumers perceived (Yan et al., 2015). Text-based consumer review analysis can capture consumers’ real emotions and evaluations of products and services. Consumers make shopping decisions through online consumer reviews (Chen and Xie, 2008; Hong et al., 2017; Li et al., 2019), and enterprises adjust their marketing strategy in response to consumer reviews (Mayzlin, 2006). Research on CBEC consumer behavior based on online consumer reviews has increased in recent years (Li et al., 2019; Wang et al., 2019), providing a new perspective on consumer behavior research (Groves, 2006; Wang Y. et al., 2018). Although several studies have investigated the influence of cultural differences on customer service quality perception, few studies have analyzed the effect of cultural differences on customer service quality cognition based on online consumer reviews. Considering that consumer reviews represent reviews of products or services from consumers from different cultures, it is important to analyze consumer perception differences in product or service quality in CBEC from the perspective of cultural differences.

This study aimed to (a) identify major service quality problems consumer encounter in cross-border e-shopping, (b) explore cultural traits affecting consumer sentiment orientation, and (c) analyze cultural traits that affect consumer service quality perception in cross-border e-shopping. To solve the above research objectives, this study applied Hofstede’s framework (Hofstede et al., 2010) as a theoretical anchor and conducted three studies. Study 1 identifies the primary service quality problems consumers are concerned about in CBEC. We extracted the service quality problems consumers perceived in cross-border e-shopping based on a latent Dirichlet allocation model (LDA) (Blei et al., 2003) and compared the differences in the service quality problems consumers are concerned about based on consumers’ cultural backgrounds. Building on the findings of study 1, study 2 analyzed the cultural influences on consumer sentiment orientation, concerning Hofstede’s six cultural dimensions, using a generalized ordered logistic regression model. Natural language processing (NLP) technology was used to infer the sentiment orientation of online consumer reviews (positive, neutral, or negative). To achieve robust results, we also control for other independent variables that can influence consumer sentiment orientation. Specifically, considering the main problems of service quality that consumers encounter (extracted from the LDA model). Building on the findings of study 1 and study 2, study 3 evaluated the effect of each cultural dimension on consumer service quality perception of cross-border e-shopping by employing a binary logistic regression model. This study provides evidence to the literature on improvements to e-commerce service quality based on cultural traits and CBEC consumer behavior studies on quality improvement. This study also has practical implications for CBEC enterprises to improve service quality from the perspective of cultural differences.

## 2. Theoretical background and hypotheses

### 2.1. Service quality, customer satisfaction, and consumer behavior

Service quality is one of the hot topics in e-commerce. Service quality is described in the context of e-commerce as the ability to support effective and efficient browsing, purchasing, and delivery of goods or services *via* a website (Zeithaml, 2000), and can be evaluated from five dimensions, including information availability and content, ease of use, privacy, graphic style, and reliability (Zeithaml et al., 2002). Based on the extensive review of existing research on electronic service quality, in the web context, Parasuraman et al. (2005) developed a scale to measure service quality according to efficiency, fulfilment, system availability, and privacy. Bauer et al. (2006) developed a four transaction stages scale to measure service quality in e-commerce from the dimensions of functionality, enjoyment, process, reliability, and responsiveness. In the field of CBEC, scholars investigate the service quality of CBEC based on characteristics such as ease of use, dependability, safety, and response (Feng and Chen, 2022). With the continuous development of information technology, some researchers identify service qualities consumers pay attention to from online consumer reviews based on text mining technology (Wang et al., 2019).

Customer satisfaction is the comparison between perceived quality and expected quality (Anderson and Sullivan, 1993; Anderson et al., 1994). Researchers conducted studies on the connections between service quality and customer satisfaction and confirmed that customer satisfaction is an emotional response to a single or long-term cognitive service contact (Gounaris et al., 2010). In the context of e-commerce, consumer satisfaction was affected by the services customers encountered and the service process (Carlson and O’Cass, 2010). Chen et al. (2013) proved that service quality positively influenced customer satisfaction. Online consumer reviews are an effective approach to identifying product features affecting consumer satisfaction (You et al., 2012). The characteristics mentioned in online consumer reviews are relatively high in importance to customer satisfaction (Xu, 2020). Enterprises can incorporate customer suggestions from online reviews into their production or service processes, thereby improving service quality and increasing customer satisfaction.

Consumers’ behavior intentions are not only affected by service quality but also responded to their satisfaction (Gounaris et al., 2010). When customers experience high-quality service, they are more likely to promote the business to others and remain loyal to an online merchant (Srinivasan et al., 2002). According to Cronin and Taylor (1992), good service can increase customer satisfaction and decrease the likelihood that they will transfer vendors.

### 2.2. Cultural differences

Culture is defined as “the collective programming of the mind which distinguishes the members of one group or category of people from another” (Hofstede, 1980). With the development of economic globalization, the importance of cultural differences in multinational services has aroused extensive discussion by scholars (Patterson et al., 2006; Reimann et al., 2008). Scholars have conducted much research on the effect of cultural differences on consumer behavior. Hofstede’s framework has been regarded as the most comprehensive theory which has received strong empirical support (Laroche et al., 2004; Patterson et al., 2006; Hofstede et al., 2010; Koh et al., 2010; Engelen and Brettel, 2011; Fang et al., 2013). Compared with other national culture theories, Hofstede’s framework is a theory that has been constantly improving and developing for decades (Hofstede, 1980; Hofstede, 1984; Hofstede et al., 2010). After decades of development, the six dimensions in Hofstede’s framework were derived, namely, power distance (PDI), individualism versus collectivism (IDV), masculinity versus femininity (MAS), uncertainty avoidance (UAI), long-term versus short-term orientation (LTO), and indulgence versus restraint (IND) (Hofstede et al., 2010). PDI indicates the extent to which the less powerful members of society can accept the unequal allocation of power. IDV reveals the degree to which society prefers a flexible social structure over a strict one. MAS indicates the extent to which dominant values in society are represented by masculinity, such as achievement and accomplishment, while femininity emphasizes care for others and relationships. UAI indicates the extent to which a society tries to avoid uncertainty through actions. LTO indicates the extent to which a society exhibits a long-term perspective rather than a short-term view. IND measures how much a society allows members to enjoy life and have enjoyment without being constrained by rigid social norms.

Hofstede’s cultural dimensions represent a valuable framework for research on web analysis, advertising, and web content development (Singh et al., 2003). Based on Hofstede’s framework, researchers conducted many studies on consumer behavior from the perspective of cultural differences. Nakayama and Wan (2019) mentioned that consumer behavior is influenced by culture, and eWOM reflects this perspective. Consumers from different cultures may perceive different service qualities of the same product or service (Donthu and Yoo, 1998; Forgas-Coll et al., 2012). Many academics revealed the influence of cultural value on customer satisfaction in the study of service quality (Furrer et al., 2000; Voss et al., 2004; Reimann et al., 2008). Some studies have demonstrated the impact of cultural differences on online consumer views in the hospitality, tourism, and entertainment industry (Koh et al., 2010; Fang et al., 2013; Ayeh et al., 2016; Hong et al., 2016). In consequence, we examine consumer sentiment orientation and service quality perceptions in cross-border e-shopping in light of Hofstede’s cultural dimensions.

### 2.3. Research hypotheses

#### 2.3.1. Power distance

PDI is defined as the extent to which the less powerful members of institutions and organizations within a country expect and accept that power is distributed unequally (Hofstede et al., 2010). Countries with high PDI consider inequality among members of a society as normal (Park et al., 2014). People show great dependence on the centralization and formalization of rights and a high tolerance for the lack of autonomy (Hofstede, 1980; Hofstede, 1984). People from a culture with high rights distance are more likely to accept rights hierarchy, strict control, and vertical, top-down communication (Hofstede, 1998). Compared with customers from countries with high power distance, those from low power distance countries have higher service quality expectations and higher expectations of responsiveness and reliability from service providers (Donthu and Yoo, 1998). Compared with low-power-distance consumers, high-power-distance consumers have relatively lower reliability expectations (Dash et al., 2008). Consumers from low power distance cultures will be dissatisfied when they do not get the expected service (Stauss and Mang, 1999). When consumers are satisfied with the service quality, they will leave positive comments through online reviews (Wang W. et al., 2018). We expect that consumers from countries with high power distance will complain less than those from low power distance countries because they will accept the authority and professional knowledge of CBEC enterprises and have lower service quality expectations for the same product or service. Thus, this study proposes the following hypothesis:

*H1*: Power distance has a positive effect on consumer sentiment orientation in online consumer reviews.

#### 2.3.2. Individualism versus collectivism

Individualism is exemplified in societies where the ties between individuals are loose, and collectivism is in those where people from birth onward are integrated into strong, cohesive in-groups (Hofstede et al., 2010). Individualistic customers prioritize their benefits, do not tolerate low service quality, and are likely to have high service quality expectations (Donthu and Yoo, 1998; Furrer et al., 2000; Dash et al., 2008; Kim et al., 2014). By contrast, collectivist customers want to have good relationships with service providers. Customers from a collectivist culture are easy to satisfy, tolerate poor services, and have no high expectations of quality of service (Donthu and Yoo, 1998; Kassim and Abdullah, 2010). Customers from collective cultures are less likely than those from individualistic cultures to leave extremely bad evaluations (Koh et al., 2010). But customers from individualistic cultures are more inclined to complain when they experience subpar customer service (Liu et al., 2001), and relatively leave negative online reviews (Mariani and Predvoditeleva, 2019). Therefore, individualistic consumers tend to complain more often about CBEC service quality than collectivist consumers. Thus, we propose:

*H2*: Individualism has a negative effect on consumer sentiment orientation in online consumer reviews.

#### 2.3.3. Masculinity versus femininity

Masculinity and femininity represent the dominant sex role patterns in the majority of both traditional and modern societies. Men are expected to be assertive, tough, and focused on material success, whereas women are supposed to be more modest, tender, and concerned with the quality of life (Hofstede et al., 2010). A society with values of masculinity often evokes self-confidence and cares less about others’ feelings (Hofstede, 1980). Compared to feminine cultures, consumers from masculine cultures are more likely to complain after purchases, more willing to express their comments on products, and express their opinions in a more aggressive way (Lam et al., 2009; Mariani and Predvoditeleva, 2019; Filieri and Mariani, 2021). Moreover, consumers from feminine (masculine) cultures are more inclined to provide a positive (negative) evaluation of their experience (Crotts and Erdmann, 2000; Fang et al., 2013). Therefore, we expect consumers from masculine cultures to express more criticism about CBEC service than consumers from feminine cultures. Thus, we propose:

*H3*: Masculinity has a negative effect on consumer sentiment orientation in online consumer reviews.

#### 2.3.4. Uncertainty avoidance

Uncertainty avoidance is defined as the extent to which the members of a culture feel threatened by ambiguous or unknown situations (Hofstede et al., 2010). It indicates the way people respond to uncertainties in life. People from societies with low uncertainty avoidance tend to accept uncertainty without much discomfort, take risks easily, and show tolerance for opinions and behaviors different from their own (Donthu and Yoo, 1998). Meanwhile, people from societies with high uncertainty avoidance have a strong need for consensus and clarity and avoid less tolerant and unclear situations. Given careful planning and risk aversion in decision-making, consumers with a high degree of uncertainty avoidance may have high service quality expectations compared with those with a low degree of uncertainty avoidance (Donthu and Yoo, 1998). Customers from cultures with a high level of uncertainty avoidance may be more likely to get disappointed if the service is not what they expected (Reimann et al., 2008). However, consumers with lower uncertainty avoidance show a higher tolerance for poor service (Voss et al., 2004; Ladhari et al., 2011). The higher the degree of uncertainty, the less satisfied customers are with service defects (Reimann et al., 2008; Huang and Crotts, 2019), which results in negative online evaluations (Mariani and Predvoditeleva, 2019). Therefore, we expect customers with a high degree of uncertainty avoidance to express greater dissatisfaction with CBEC services, and propose:

*H4*: Uncertainty avoidance has a negative effect on consumer sentiment orientation in online consumer reviews.

#### 2.3.5. Long-term versus short-term orientation

Long-term orientation refers to the fostering of virtues oriented toward the future re-wards-particularly perseverance and thrift. Its opposite, short-term orientation, refers to the fostering of virtues related to the past and present—particularly respect for tradition, preservation of “face,” and fulfilment of social obligations (Hofstede and Bond, 1998; Hofstede et al., 2010). Customers from countries with a long-term orientation do not eagerly pursue the truth (Hofstede, 1980). Consumers with a high degree of long-term orientation accept changes and pursue tranquility (Zhang et al., 2020). They can accept poor services and will not excessively pursue the perfection of services. They are willing to give service providers time to improve service quality (Donthu and Yoo, 1998). Consumers from countries with long-term orientation complain less than those from short-term orientation countries. When experiencing good service, they willingly praise service providers and share their experiences with others (Liu et al., 2001; Liu, 2006). Customers who have a high level of long-term orientation like to post more positive and beneficial online reviews to help service providers improve their services (Kwok and Xie, 2016). Thus, we expect the former to complain less about CBEC service quality than the latter. Thus, we propose:

*H5*: Long-term orientation has a positive effect on consumer sentiment orientation in online consumer reviews.

#### 2.3.6. Indulgence versus restraint

Indulgence refers to a tendency to allow relatively free gratification of basic and natural human desires related to enjoying life and having fun. Its opposite, restraint, refers to a conviction that this type of gratification needs to be curbed and regulated by strict social norms (Hofstede et al., 2010). Indulgence versus restraint has a significant impact on consumer desires and decision-making processes (Heydari et al., 2021). Customers from indulgent cultures are more expressive and eager to write in-depth online reviews (Filier et al., 2019; Leon, 2019; Filier et al., 2020), as well as more satisfied with the quality of the services they receive (Huang and Crotts, 2019). In an indulgent society, more people show extraversion, and fewer people show neuroticism. Extroversion relates to positive emotions, while neuroticism refers to the tendency to experience negative emotions (Hofstede et al., 2010). We expect consumers from indulgent cultures to be more likely to express positive emotions about CBEC service than consumers from restrictive cultures, and propose:

*H6*: Indulgence has a positive effect on consumer sentiment orientation regarding online consumer reviews.

## 3. Materials and methods

### 3.1. Research subjects

Aliexpress, one of China’s largest cross-border export B2C platforms, with consumers from more than 220 countries and regions, was selected as the data source for this paper. This study takes cell phones as the research object. The object is considered reasonable because (1) mobile communication products are considered the “advantage” and top-selling products on the Aliexpress e-commerce platform, (2) they have a high rate of online consumer reviews, (3) different from other popular products such as women’s fashion or accessories, cell phones are general goods that are less affected by consumer cultural traits.

### 3.2. Analyzing framework

Taking cell phones as the research object, this study identified the main issues that consumers pay attention to in the process of cross-border shopping, analyzed the influence of cultural traits on the sentiment of online reviews, and explored the impact of cultural differences on consumers’ perception of cross-border e-commerce service quality.

First, we collected online reviews, store information, and phone information from AliExpress.com. The LDA topic model is used to extract hidden topics (i.e., the main quality service problems that consumers pay attention to in cross-border shopping) in cell phone online reviews. We then calculate the topic intensity, based on which we compare differences among consumers in nine countries in terms of their level of concern for these service quality problems. We use NLP technology to evaluate the reviews and analyze the influence of culture on consumer sentiment orientation. TextBlob (a python library for processing English textual data) is used to process the reviews and predict the probability that a review is positive, neutral, or negative.

Second, we examined the influence of cultural traits on consumers’ sentiment orientation based on Hofstede’s cultural dimensions by employing a generalized ordered logistic regression model. Since consumer satisfaction is also affected by other factors (Wang et al., 2019). Therefore, we also account for other independent variables that may have an impact on consumer sentiment orientation, which represent the main service quality issues consumers are concerned about during cross-border shopping (extracted from the LDA model).

**Figure 1:**
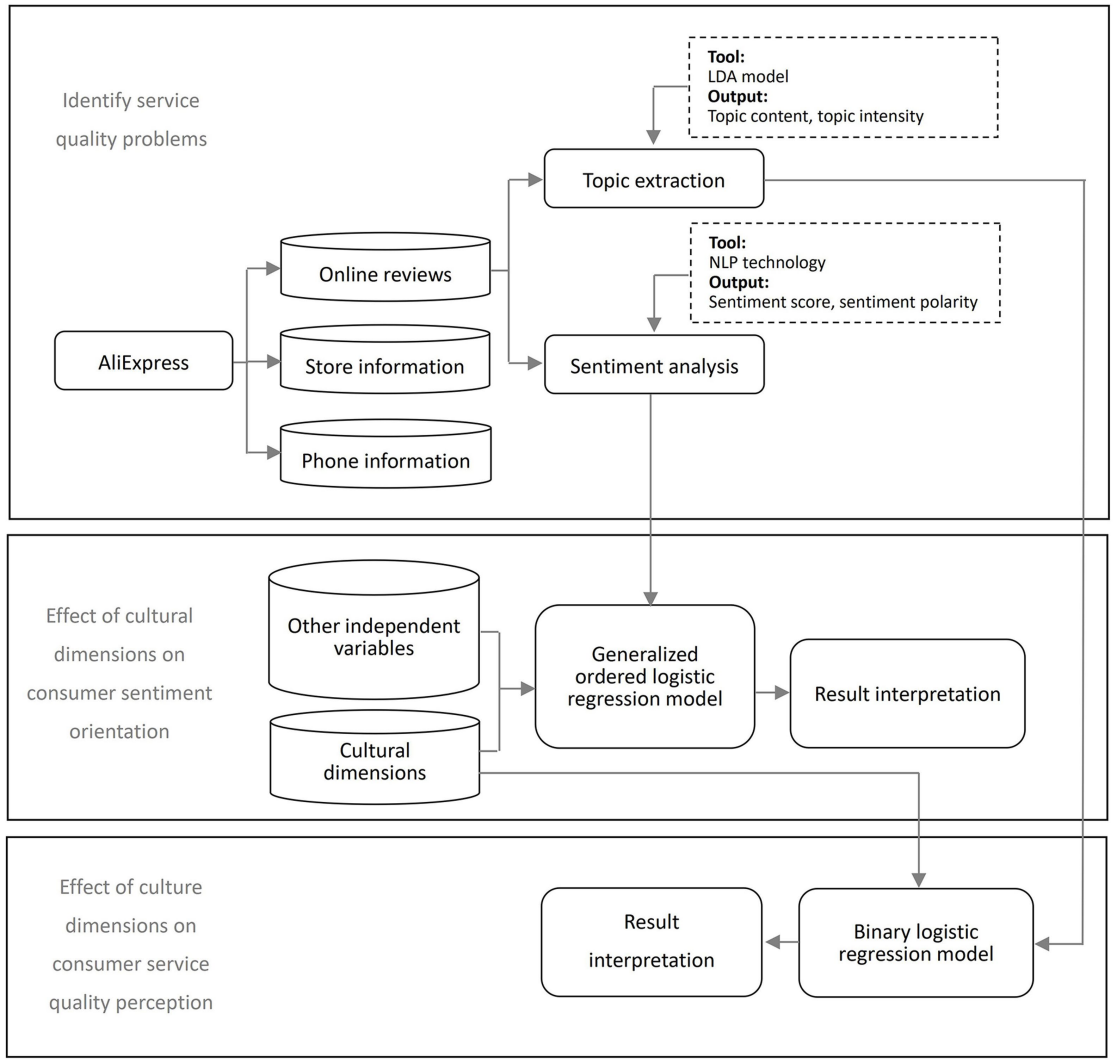
Research framework.

Finally, the effects of the cultural dimensions on consumer service quality perceptions were estimated based on a binary logistic regression model. Using the median of each topic probability as a threshold, we divide each document’s document-subject probability into strong and weak states to produce a multi-dimensional vector representing customer concerns about each topic, which are scored on a two-point scale (1 denotes strong and 0 denotes weak). The research framework is shown in Figure 1.

### 3.3. Study 1

#### 3.3.1. Data collection and pre-processing

Aliexpress displays mobile communication products by brand and price. According to the product price intervals, we select mobile phone models with high orders in different price intervals. The selected cell phone product types cover all the bestsellers from high- to low-end phones. We write crawlers to collect cell phone product user reviews from January 2019 to December 2019, store attributes, and phone attributes. The collected user review data include the user’s country and review content. The store attribute data included the score of the store in items as described, shipping speed, and communication. Phone attribute data include mobile phone price, brand, battery capacity, CPU, and so on. The user comment, store attributes, and phone attribute datasets are correlated by the product ID data. Because the number of cell phone user reviews in some countries is small, we select the top nine countries as the research target. Finally, we selected user reviews from nine countries containing 487 phone models with complete store and product attributes.

Scientific online consumer reviews have a specific guiding influence on consumer consumption views and behaviors. Hence, some sellers tend to post false positive reviews of their products. On Aliexpress, only consumers who have purchased goods have the right to comment on their shopping experience. Aliexpress has strict review management rules. If the reputation of a store is questionable, Aliexpress will delete the shipment and other irregularities. False reviews rarely involve detailed descriptions of product quality and customer experience, resulting in shorter comments (Zhang, 2019). Therefore, we delete duplicate reviews and user reviews of less than five words for the same product from the same country consumer and finally got 59,736 English user reviews from consumers in nine countries. Table 1 shows the number of user reviews in each country.

**Table 1:** Number of reviews by country.

Country	Number of reviews	Country	Number of reviews
Russia	29,590	United States	2,524
Brazil	13,234	France	1,802
Algeria	3,949	Kazakhstan	1,286
Spain	3,373	Poland	1,154
Mexico	2,824		
In total	59,736		

#### 3.3.2. Measures

The LDA model is used to mine the service issues that consumers mainly focused on in cross-border e-commerce shopping. The LDA model is proved to be an efficient topic model to extract abstract topics from text data (Li et al., 2019; Mou et al., 2019; Kang et al., 2022).

In LDA model, a corpus is a collection of *D* documents, a document is a sequence of *N* words, and *K* is the number of topics. $z_{n}$ is the topic for the $n_{th}$ word in document *d*. $\theta_{d}$ is topic distribution for document *d*. $\varphi_{k}$ is word distribution for topic *k*.

The basic idea is that the documents are represented as a random blend of latent topics, where each topic is characterized by a distribution of words. Each document d is generated as follows:

- Choose N~Poisson(ξ) .
- Choose $\theta_{d}\sim \mathrm{Dirichlet}\left(\alpha \right)$ .
- For each of the *N* words $w_{n}$ :
  - Choose a topic $z_{n}\sim \mathrm{Multionmial}\;\left(\theta_{d}\right)$ .
  - Choose a word $w_{n}$ from $p\left(w_{n}|z_{n},\beta \right)$ , a multinomial probability conditioned on the topic $z_{n}$ .

**Figure 2:**
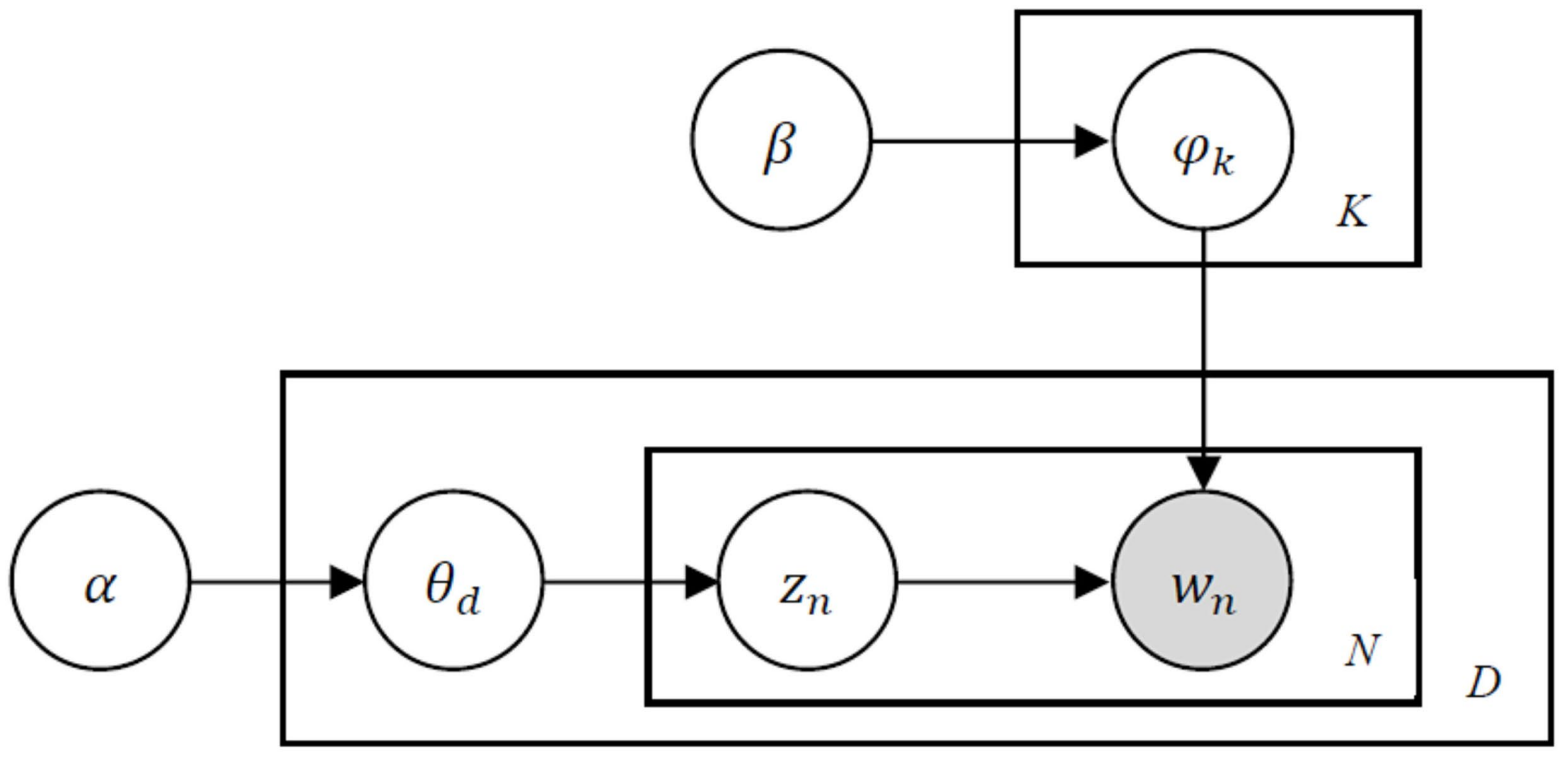
Graphical representation of LDA.

The graphical representation of LDA is shown in Figure 2.

The word generation process of each document is as follows:


(1) $$p\left(\theta ,z,w|\alpha ,\beta \right)=p\left(\theta |\alpha \right)\;\overset{N}{\underset{n=1}{\prod }}p\left(z_{n}|\theta \right)\;p\left(w_{n}|z_{n},\beta \right)$$

The marginal distribution function is calculated as follows:


(2) $$p\left(w|\alpha ,\beta \right)=\int_{}p\left(\theta |\alpha \right)\;\overset{N}{\underset{n=1}{\prod }}p\left(z_{n}|\theta \right)\;p\left(w_{n}|z_{n},\beta \right)d_{\theta }\;$$

Given the parameters α and β , the joint distribution is given by:


(3) $$\begin{array}{l} p\left(D|\alpha ,\beta \right)=\overset{\left|D\right|}{\underset{d=1}{\prod \;}}\;\;\;\;\;\;\;p\left(\theta_{d}|\alpha \right)\;\\ \;\;\;\;\;\;\;\;\;\;\;\;\;\;\;\;\;\;\left(\overset{N_{d}}{\underset{n=1}{\prod }}\;\;\;\;\;\;\;\;\underset{z_{d_{n}}}{\sum }\;\;\;\;\;\;p\left(z_{d_{n}}|\theta_{d}\right)p\left(w_{d_{n}}|z_{d_{n}},\beta \right)d\theta_{d_{n}}\right) \end{array}$$

This study selected the Python Gensim toolkit to train and generate the LDA model. To improve the accuracy, we took the following steps to pre-process the data for the 59,736 collected comments: (1) conversion of uppercase letters to lowercase letters and removal of punctuation and special symbols, (2) segmentation of words, (3) identification of stop words, and (4) removal of low-frequency words. Next, we created a word dictionary, generated a document–term matrix from the word dictionary, and then trained the LDA model. The probability vector of topic-document was obtained through the LDA model.

To analyze consumers’ attention to each topic, we calculated the intensity of each topic according to the following formula (Griffiths and Steyvers, 2004):


(4) $$\theta_{z}=\frac{\sum_{d=1}^{D}\theta_{z}^{d}}{D}$$

Where， $\theta_{z}$ is the intensity strength of topic *z* (*z* = 1, …, *K*), $\theta_{z}^{d}$ is the probability of document *d* (*d* = 1,..., *D*) generated by topic *z*. In order to ensure a clear boundary between topics, feature words (e.g., “phone,” “product,” etc.) that are unclear and appear on multiple topics were deleted. Finally, 10 words were selected as high-frequency feature words, and which are arranged in descending order.

### 3.4. Study 2

#### 3.4.1. Data collection and pre-processing

Most e-commerce websites require users to provide a digital rating of 1–5 stars for service quality when they make comments. For example, one star represents “very dissatisfied,” while five stars represent “very satisfied.” Many scholars have evaluated the emotion of user reviews according to user ratings. Many sellers on Aliexpress have received praise from consumers for supplying gifts and coupons. Therefore, to obtain additional benefits, consumers tend to give sellers five stars to praise them, but consumers express their real shopping experience in written comments.

To explore cultural traits affecting consumer sentiment orientation, this study used NLP technology to analyze the emotional tendencies of CBEC consumers based on the online consumer reviews collected in study 1. The specific steps are as follows. First, we used TextBlob to analyze the sentiment of CBEC consumer reviews. We obtained a polarity score between −1 (meaning negative) and 1 (positive). Second, based on the emotional score of user reviews, we labeled user reviews as less than 0 (meaning negative), greater than 0.5 (positive), and others (neutral). Eventually, we got 33,301 positive reviews, 18,107 neutral reviews, and 8,328 negative reviews.

#### 3.4.2. Measures

This study examined the effect of cultural traits on consumer sentiment orientation based on Hofstede’s cultural dimensions by employing a generalized ordered logistic regression model. This regression model relaxes the parallel line hypothesis and suits models that are less restrictive than ordered logistic regression models (Williams, 2006). Consumer satisfaction is also affected by other factors (Wang et al., 2019). Therefore, we add to the model other measurable variables that could affect consumer satisfaction, based on the results of the topic analysis. The data for these variables are mainly derived from the relevant indicators of the countries where consumers are located, as well as store and phone attribute information.

The model is based on the relationship between consumer satisfaction and cultural differences. We define an ordered variable as the dependent variable, where =1, = 0, and = −1 if the sentiment analysis result of the *i*th review record was positive, neutral, and negative, respectively. Hofstede’s six cultural dimensions are the independent variables. According to the topic analysis results, some topics can be expressed as measurable variables.

We formulated a generalized ordered logistic regression to estimate the impact of the aforementioned variables on ${\mathrm{satisfaction}}_{i}$ as follows:


(5) $$P\left({\mathrm{satisfaction}}_{i}>j\right)=g\left(X\beta_{j}\right)=\frac{\exp\left(\alpha_{j}+X\beta_{j}\right)}{1+\exp\left(\alpha_{j}+X\beta_{j}\right)}$$

Where the number of categories of the ordinal dependent variable is 3, and *j* = 1, 2. *X* is a set of independent variables. $\beta_{j}$ is the set of independent variable coefficients. From the above, it can be determined that the probabilities that satisfaction take on each of the values 1, and 2 are equal to:


(6) $$P\left({\mathrm{satisfaction}}_{i}=j\right)=1-g\left(X\beta_{1}\right),j=1$$

(7) $$P\left({\mathrm{satisfaction}}_{i}=j\right)=g\left(X\beta_{j-1}\right)-g\left(X\beta_{j}\right),j=2\;$$

### 3.5. Study 3

#### 3.5.1. Data collection and pre-processing

To evaluate the effect of each cultural dimension on consumer service quality perception of cross-border e-shopping, building on the results of study 1, according to Equation (8), we transform the document-topic probability of each online consumer review into strong and weak states using the median of each topic probability as a threshold and obtain a multi-dimension vector of consumer concerns about each topic, which is measured on a two-point scale (1 denotes strong and 0 denotes weak).


(8) $$X_{ij}=\{\begin{array}{c} 1,if\;p_{ij}>\mathrm{MEDIAN}\left(p_{1j,}p_{2j,}\cdots p_{59736j}\right)\\ 0,if\;p_{ij}\leq \mathrm{MEDIAN}\left(p_{1j,}p_{2j,}\cdots p_{59736j}\right) \end{array}\;$$

Where $p_{ij}$ represents the probability value of the ith (*i* = 1, …, 59,736) review belongings to topic *j* (*j* = 1, …, *K*). As a result, we obtain a *K*-dimensional vector of consumer concerns on the *K* service quality problems in cross-border e-shopping (see Table 2):

**Table 2:** Structure data of the main service quality problems that consumer concerned.

	Topic			
Review	$X_{\mathrm{ij}}$ (*j* = 1)	$X_{\mathrm{ij}}$ (*j* = 2)	…	$X_{\mathrm{ij}}$ (*j* = *K*)
*i* = 1	1	1	…	0
*i* = 2	0	1		0
…	…	…	…	…
*i* = 59,736	1	0		1

#### 3.5.2. Measures

A binary logistic regression model is explored to measure the influence of cultural dimensions on consumer perception of service quality in CBEC.

The *K*-dimensional vector and the values of the six cultural dimensions are, respectively, constructed into *K* data sets. We define $X^{j}$ (*j* = 1, …, *K*) as dependent variables and the six cultural dimensions as independent variables. We formulated K binary logistic regression models as follows:


(9) $$\begin{array}{l} X^{j}=\alpha +\beta_{j\_1}PDI+\beta_{j\_2}IDV+\\ \;\;\;\;\;\;\;\;\;\beta_{j\_3}MAS+\beta_{j\_4}UAI+\beta_{j\_5}LTO+\beta_{j\_6}IND \end{array}$$

Where, $X^{j}$ is the jth (*j* = 1, …, *K*) service quality problem that consumers concerned with.

## 4. Results

### 4.1. Study 1

By considering the literature (Li et al., 2019; Mou et al., 2019; Wang et al., 2019) on the determinants of mobile communication product service quality in CBEC, referring to the expert opinions of CBEC enterprises, and calculating the degree of confusion. This study determined 14 as the optimal number of topics, and the probability vector of the topic-document was obtained through the LDA model. The intensity and high-frequency feature words of each topic are shown in Table 3.

**Table 3:** Topic names, topic intensity and example words with high probabilities.

Topic number	Topic name	Topic intensity	Example words with high probabilities
1	Shipping speed	0.0109	Seller, time, long, order, sent, day, month, delivery, week, received
2	Gifts	0.0048	Glass, film, cover, case, protective, silicone, screen, adapter, headphone, charger
3	Cell phone version	0.0087	New, version, came, ordered, global, Chinese, review, cool, later, add
4	Item as described	0.0039	Description, corresponds, beautiful, color, look, good, according, characteristic, match, declared
5	Cell phone performance	0.0026	Note, gb, properly, test, 8, bought, 6, show, loved, 128
6	Seller recommendation	0.0079	Excellent, great, fast, recommend, seller, quality, price, liked, device, nice
7	Customs efficiency and tariff	0.0127	Arrived, day, taxed, good, well, perfect, condition, fast, took, came
8	Cell phone function	0.0085	Camera, battery, good, work, screen, use, well, charge, normal, sound
9	Trust in sellers	0.0036	Store, shipping, buy, service, far, hope, definitely, first, impression, reliable
10	Logistics service	0.0145	Everything, came, day, seller, delivery, quickly, work, fine, packed, fast
11	Value for money	0.0051	Price, best, without, custom, buy, cost, value, problem, paid, purchase
12	Logistics package quality	0.0023	Perfectly, packed, working, normally, top, work, equipment, flight, solid, fall
13	Customs dispute	0.0054	Custom, money, pay, seller, post, dispute, office, parcel, opened, duty
14	Shopping experience	0.0089	Good, thank, seller, happy, everything, satisfied, received, fast, ok, purchase

A stronger representation of the topic intensity column in bold.

Based on the Equation (4), we divided the 14 topic probability values corresponding to each country’s user reviews and the total number of user reviews. Finally, 14 thematic intensity values of these nine countries were obtained. By comparison the intensity of these 14 themes, the differences in the intensity of consumer reviews in nine countries were analyzed. Table 4 shows that the consumers in this study paid more attention to the service quality of logistics service, customs efficiency and tariff, shipping speed, shopping experience, cell phone function, cell phone version, and seller recommendation in cross-border e-shopping. Compared with other countries, consumers in Russia, the United States, and Kazakhstan were most concerned about logistics service. Consumers in Brazil, Spain, Mexico, and France were most interested in customs efficiency and tariff. Lastly, Algerian (Polish) consumers were most concerned about the shopping experience (shipping speed).

**Table 4:** Topic intensity by country.

Topic number	Topic name	Topic intensity								
		RU	BR	DZ	ES	MX	US	FR	KZ	PL
1	Shipping speed	0.123	0.062	0.133	0.128	0.122	0.099	0.116	0.133	**0.164**
2	Gifts	0.061	0.031	0.027	0.045	0.033	0.043	0.041	0.060	0.042
3	Cell phone version	0.120	0.040	0.038	0.061	0.056	0.077	0.062	0.126	0.083
4	Item as described	0.035	0.048	0.039	0.036	0.033	0.042	0.047	0.040	0.044
5	Brand and cell phone performance	0.024	0.033	0.018	0.026	0.033	0.026	0.028	0.023	0.024
6	Seller recommendation	0.063	0.112	0.092	0.063	0.113	0.091	0.079	0.051	0.059
7	Customs efficiency and tariff	0.040	**0.296**	0.193	**0.166**	**0.181**	0.122	**0.127**	0.044	0.075
8	Cell phone function	0.094	0.069	0.047	0.104	0.061	0.095	0.110	0.089	0.104
9	Trust in sellers	0.028	0.041	0.052	0.044	0.061	0.043	0.040	0.033	0.047
10	Logistics service	**0.220**	0.049	0.059	0.068	0.070	**0.124**	0.080	**0.203**	0.101
11	Value for money	0.036	0.077	0.048	0.067	0.062	0.055	0.059	0.035	0.064
12	Logistics package quality	0.021	0.027	0.018	0.022	0.027	0.023	0.024	0.020	0.019
13	Customs dispute	0.051	0.045	0.035	0.089	0.065	0.059	0.064	0.074	0.115
14	Shopping experience	0.083	0.071	**0.202**	0.081	0.084	0.103	0.125	0.068	0.060

*The maximum topic intensity value for each country is marked in bold*. RU, Russia; BR, Brazil; DZ, Algeria; ES, Spain; MX, Mexico; US, United States; FR, France; KZ, Kazakhstan; PL, Poland.

### 4.2. Study 2

We define an ordered variable, ${\mathrm{satisfaction}}_{i}$ , as the dependent variable, Hofstede’s six cultural dimensions are the independent variables. Based on the topic analysis results, some topics can be expressed as measurable variables, such as customs efficiency and tariff, item as description, trust in sellers, shipping speed, seller recommendation, cell phone function, gifts, value for money, cell phone performance, and cell phone function.

These variables can also affect consumer satisfaction (Li et al., 2019; Wang et al., 2019); hence, we include them in the model as independent variables. These independent variables are (i) detailed seller ratings (*ItemAsDescribed*, *Communication*, *ShippingSpeed*), (ii) cell phone attributes (*Price, Battery, ROM, Screen, Finger, Gifts, CPU, Brand*), and (iii) customs efficiency and tariff of the importing countries (*Customsefficiency&tariff*). The variable data of detailed seller ratings and cell phone attributes were collected from the dataset of store information and phone information. Variable data on *Customsefficiency&tariff* were collected from the 2019 Global Competitiveness Report. According to the index calculation method in the Global Competitiveness Report, the mean value of the sum of border clearance efficiency and trade tariff was taken as the value of the variable *Customsefficiency&tariff*. The cultural dimension values collected from the Hofstede-insights website^1^ are based on the consumer country information shown in online consumer reviews. Table 5 describes in detail the independent variables in the model.

**Table 5:** Description of Independent variable.

Variable	Description	Summary	Data source
PDI	Power distance	0–100 (out of 100)	Hofstede-insights
IDV	Individualism vs. Collectivism	0–100 (out of 100)	Hofstede-insights
MAS	Masculinity vs. Femininity	0–100 (out of 100)	Hofstede-insights
UAI	Uncertainty Avoidance	0–100 (out of 100)	Hofstede-insights
LTO	Long-Term vs. Short-Term Orientation	0–100 (out of 100)	Hofstede-insights
IND	Indulgence vs. Restraint	0–100 (out of 100)	Hofstede-insights
*Customsefficiency&tariff*	The index value of border clearance efficiency and customs tariff	26.4–79.2 (out of 100)	The Global Competitiveness Report 2019
*ItemAsDescribed*	Item as described rating score from buyers in the past 6 months	2.3–5 (out of 5)	Store attributes
Communication	Communication rating score from buyers in the past 6 months	1.7–5 (out of 5)	Store attributes
*ShippingSpeed*	Shipping speed rating score from buyers in the past 6 months	1.6–5 (out of 5)	Store attributes
Battery	Battery capacity (mAh)	330–13,000	Phone attributes
ROM	The ROM storage (G)	0.27–512	Phone attributes
Screen	Size of screen (inch)	2–7.2	Phone attributes
Finger	Whether finger recognition is available	Support, not support	Phone attributes
CPU	Number of CPU cores	Quad core, Octa core, etc.	Phone attributes
Brand	Phone brand	Oneplus, Xiaomi, etc.	Phone attributes
Price	Phone price (USD)	11–937	Phone attributes
Gifts	Whether gifts are available	Support, not support	Phone attributes

$Customsefficiency\;\&tariff=\left(\mathrm{border clearance efficiency}+\mathrm{customs tariff}\right)/2$ , the value of border clearance efficiency and customs tariff come from the Global Competitiveness Report 2019.

We adjusted the attribute values of some independent variables in the model. The *CPU* attribute values of Dual core, Quad core, Hexa core, Octa-core, and Deca core were set to 1, 2, 3, 4, and 5 according to the CPU performance. There are 47 cell phone brands such as Oneplus, UMIDIGI, Meizu, Xiaomi, Oukitel, Blackview, and so on. If the number of online consumer reviews of the brand is less than 1,000, the brand attribute value is set to 0 and 1 otherwise. If the cell phone contains gifts, the value of *Gifts* is set to 1 and 0 otherwise. If the phone has a fingerprint recognition function, the value of *Finger* is set to 1 and 0 otherwise.

Table 6 summarizes the results of estimating the model based on the sample of 59,736 reviews. The regression results show that PDI has a positive effect on consumer satisfaction. In the negative and neutral panels, the coefficients for PDI are 0.135 (*p* < 0.01) and 0.070 (*p* < 0.01), thus supporting H1. In the negative panel, the coefficient for IDV shows a significant negative effect, but in the neutral panel, IDV shows a significant positive effect; therefore, H2 is partially supported. In the negative and neutral panels, the coefficients for UAI are −0.336 (*p* < 0.01) and − 0.148 (*p* < 0.01), meaning that UAI negatively affects consumer satisfaction, thus supporting H4. In the negative panel, the coefficients for MAS and LTO show a significant positive effect, but in the neutral panel, MAS and LTO show a significant negative effect; therefore, H3 and H5 are partially supported. In the negative and neutral panels, the coefficients for IND are 0.076 (*p* < 0.05) and 0.630 (*p* < 0.01) respectively, thus supporting H6.

**Table 6:** Description of independent variable.

Variable	Negative	Neutral	Variable	Negative	Neutral
	Coef(SE)	Coef(SE)		Coef(SE)	Coef(SE)
Constant	1.449***	−0.081	*ShippingSpeed*	0.041	0.013
	(0.081)	(0.062)		(0.026)	(0.019)
PDI	0.135***	0.070***	ROM	−0.160***	−0.115***
	(0.029)	(0.022)		(0.012)	(0.010)
IDV	−0.127***	0.072***	Screen	0.062***	0.087***
	(0.027)	(0.020)		(0.015)	(0.012)
MAS	0.121***	−0.547***	Finger	0.131***	0.089***
	(0.024)	(0.021)		(0.032)	(0.023)
UAI	−0.336***	−0.148***	Gifts	−0.027	−0.040
	(0.029)	(0.024)		(0.034)	(0.025)
LTO	0.290***	−0.132***	Price	0.020	−0.025***
	(0.035)	(0.028)		(0.014)	(0.009)
IND	0.076**	0.630***	Brand	0.099**	0.073**
	(0.030)	(0.028)		(0.040)	(0.031)
*Customesfficiency&tariff*	−0.119***	−0.209***	CPU	0.062***	0.052***
	(0.020)	(0.015)		(0.018)	(0.014)
*ItemAsDescribed*	0.152***	0.104***	Battery	−0.030**	−0.079***
	(0.023)	(0.018)		(0.013)	(0.009)
Communication	−0.065**	−0.022			
	(0.030)	(0.022)			

Standard errors in parenthesis. ***p < 0.01, **p < 0.05.

*ItemasDescribed* has significant positive effects on consumer satisfaction. The higher the *ItemasDescribed* score of the seller, the more likely consumers are to leave positive online consumer reviews. In the negative panel, the coefficient for *Communication* is −0.065 (*p* < 0.05), but in the neutral panel, the coefficient for *Communication* is not significant, which means a higher *Communication* score of the seller may lead to consumers’ negative sentiment, but may not promote consumer positive sentiment. The reason may be that consumers who make orders from stores with high *Communication* scores have high service communication expectations, while CBEC is often affected by uncertainty factors such as logistics, customs efficiency and tariff, such that sellers may fail to respond clearly and accurately, which leads to negative sentiment. Excellent communication is a basic service requirement for consumers but is not an important service to improve consumer satisfaction.

In the negative and neutral panels, the coefficient for *Customsefficiency&tariff* is −0.119 (*p* < 0.01) and −0.209 (*p* < 0.01). Consumers who come from countries with higher *Customesfficiency&tariff* scores likely will not be satisfied with the service. The reason may be that consumers who come from countries with low *Customesfficiency&tariff* scores may have lower custom tariff and border clearance efficiency expectations. Consumer satisfaction is not easily affected by high trade tariffs or low border clearance efficiency, which can be verified by online consumer reviews from countries with low *Customesfficiency&tariff* scores such as Brazil and Mexico (Table 7).

**Table 7:** Sample reviews from Brazil and Mexico.

Country	*Customesfficiency & tariff*	Sample review
Brazil	26.45	Excellent product. Fast shipping, the problem is the Brazilian customs
Brazil	26.45	Seller and meets the agreed. Product arrived late, due to slow customs and postal system
Brazil	26.45	Excellent enjoyed great seller The Brasil problem and the post office and customs delays that always deliveries
Mexico	55	Arrived in perfect condition, the delay was the custom house, 55 days of purchase to get
Mexico	55	Excellent Product, quality price but if you are from Mexico please note it will be withheld by customs and charge you 780 extras
Mexico	55	They arrived safely, the only problem is that for one I had to pay $ 1699 Mexican pesos, customs responsibility and their raffle

In the negative and neutral panels, the coefficient for *Battery* and *ROM* are significantly negative, indicating that the larger the battery capacity and ROM of the cell phone, the more likely these will cause consumers’ negative emotions, which is likely related to consumers’ high expectations of battery and ROM performance. In the negative and neutral panels, the coefficient for *Screen*, *brand*, and *CPU* are significantly positive, indicating that *Screen*, *brand*, and *CPU* significantly positively promote consumer satisfaction. *Finger* significantly positively affects consumer satisfaction, which means that cell phones with a finger recognition function may lead to positive sentiment.

In the negative panel, the coefficient for *Price* is not significant, while in the neutral panel, the coefficient is significantly negative, indicating that relative to negative emotions, *Price* cannot significantly affect consumers’ neutral and positive emotions, but relative to negative and neutral emotions, *Price* negatively affects consumers’ positive emotions. *Gifts* cannot significantly affect consumers’ satisfaction.

### 4.3. Study 3

According to the results of study 1, we obtained a 14-dimensional vector of consumer concerns on the 14 service quality problems in cross-border e-shopping and formulated 14 binary logistic regression models. To analyze the influence of culture on consumer concerns regarding different service quality problems, we horizontally compare the coefficients for each of Hofstede’s cultural dimensions in 14 models (see Table 8).

**Table 8:** Results of binary logistic regression model.

Variable	PDI		Variable	IDV		Variable	MAS		Variable	UAI		Variable	LTO		Variable	IND	
	Coef (SE)	OR		Coef (SE)	OR		Coef (SE)	OR		Coef (SE)	OR		Coef (SE)	OR		Coef (SE)	OR
*β*_1-1_	0.133***	1.142	*β*_1-2_	0.140***	1.15	*β*_1-3_	0.089***	1.095	*β*_1-4_	0.196***	1.222	*β*_1-5_	−0.355***	0.70	*β*_1-6_	−0.257***	0.77
	(0.021)			(0.014)			(0.018)			(0.019)			(0.022)			(0.023)	
*β*_2-1_	0.063***	1.07	*β*_2-2_	0.038***	1.04	*β*_2-3_	−0.069***	0.937	*β*_2-4_	−0.063***	0.943	*β*_2-5_	0.012	1.01	*β*_2-6_	−0.0466**	0.96
	(0.021)			(0.014)			(0.018)			(0.019)			(0.021)			(0.022)	
*β*_3-1_	0.213***	1.26	*β*_3-2_	0.168***	1.18	*β*_3-3_	0.179***	1.20	*β*_3-4_	−0.014	0.998	*β*_3-5_	0.167***	1.18	*β*_3-6_	−0.227***	0.80
	(0.021)			(0.014)			(0.018)			(0.019)			(0.021)			(0.023)	
*β*_4-1_	−0.031	0.971	*β*_4-2_	−0.042***	0.96	*β*_4-3_	−0.129***	0.886	*β*_4-4_	−0.093***	0.914	*β*_4-5_	−0.151***	0.86	*β*_4-6_	−0.012	0.99
	(0.021)			(0.014)			(0.018)			(0.019)			(0.021)			(0.220)	
*β*_5-1_	−0.049**	0.956	*β*_5-2_	−0.049***	0.95	*β*_5-3_	−0.197***	0.821	*β*_5-4_	−0.037**	0.969	*β*_5-5_	−0.172***	0.84	*β*_5-6_	0.131***	1.14
	(0.021)			(0.014)			(0.018)			(0.019)			(0.021)			(0.023)	
*β*_6-1_	−0.013	0.999	*β*_6-2_	−0.076***	0.93	*β*_6-3_	−0.152***	0.865	*β*_6-4_	−0.075***	0.930	*β*_6-5_	−0.148***	0.86	*β*_6-6_	0.238***	1.27
	(0.021)			(0.014)			(0.018)			(0.019)			(0.021)			(0.023)	
*β*_7-1_	−0.645***	0.538	*β*_7-2_	−0.470***	0.63	*β*_7-3_	−0.424***	0.650	*β*_7-4_	−0.033	0.979	*β*_7-5_	−0.200***	0.82	*β*_7-6_	0.512***	1.67
	(0.023)			(0.014)			(0.019)			(0.020)			(0.023)			(0.024)	
*β*_8-1_	−0.065***	0.941	*β*_8-2_	0.070***	1.07	*β*_8-3_	−0.034*	0.975	*β*_8-4_	0.047***	1.059	*β*_8-5_	0.006	1.00	*β*_8-6_	−0.046**	0.96
	(0.021)			(0.014)			(0.018)			(0.019)			(0.021)			(0.022)	
*β*_9-1_	0.004	1.005	*β*_9-2_	−0.008	0.99	*β*_9-3_	−0.086***	0.925	*β*_9-4_	0.052**	1.059	*β*_9-5_	−0.346***	0.711	*β*_9-6_	0.022	1.02
	(0.021)			(0.014)			(0.018)			(0.019)			(0.021)			(0.023)	
*β*_10-1_	0.510***	1.67	*β*_10-2_	0.275***	1.32	*β*_10-3_	0.264***	1.30	*β*_10-4_	−0.174***	0.849	*β*_10-5_	0.456***	1.56	*β*_10-6_	−0.269***	0.76
	(0.023)			(0.014)			(0.018)			(0.020)			(0.022)			(0.023)	
*β*_11-1_	−0.252***	0.782	*β*_11-2_	−0.090***	0.91	*β*_11-3_	−0.120***	0.891	*β*_11-4_	0.102***	1.112	*β*_11-5_	−0.223***	0.80	*β*_11-6_	0.047**	1.050
	(0.021)			(0.014)			(0.018)			(0.019)			(0.021)			(0.022)	
*β*_12-1_	0.020	1.020	*β*_12-2_	−0.060***	0.94	*β*_12-3_	−0.202***	0.820	*β*_12-4_	−0.131***	0.884	*β*_12-5_	−0.150***	0.86	*β*_12-6_	0.104***	1.11
	(0.021)			(0.014)			(0.018)			(0.019)			(0.022)			(0.023)	
*β*_13-1_	−0.179***	0.847	*β*_13-2_	0.021	1.02	*β*_13-3_	−0.009	0.991	*β*_13-4_	0.230***	1.268	*β*_13-5_	−0.292***	0.75	*β*_13-6_	−0.115***	0.89
	(0.021)			(0.014)			(0.018)			(0.019)			(0.021)			(0.023)	
*β*_14-1_	0.196***	1.22	*β*_14-2_	0.063***	1.07	*β*_14-3_	−0.248***	0.786	*β*_14-4_	−0.098***	0.915	*β*_14-5_	−0.421***	0.664	*β*_14-6_	−0.029	0.97
	(0.021)			(0.014)			(0.018)			(0.019)			(0.022)			(0.023)	

Standard errors in parenthesis. ***p < 0.01, **p < 0.05, *p < 0.1, *j* = 1, …, 14. 1, Shipping speed; 2, Gifts; 3, Cell phone version; 4, Item as described; 5, Cell phone Performance; 6, Seller recommendation; 7, Customs efficiency & tariff; 8, Cell phone function; 9, Trust in sellers; 10, Logistics services; 11, Value for Money; 12, Logistics package quality; 13, Customs dispute; 14, Shopping experience.

Logistics service is greatly affected by PDI and LTO, both of which have a significant positive impact on logistics service ( $\beta_{10\_1}$ = 0.510, *p* < 0.01, $\beta_{10\_5}$ = 0.456, *p* < 0.01). A unit’s increase in PDI (LTO) leads to a 67% (56%) increase in consumers’ concern about logistics service. Table 9 shows that the values of PDI and LTO are high in Kazakhstan, and Russia. As Table 4 illustrates, the topic intensity value of logistics service in Kazakhstan, and Russia is higher than those of other topics.

**Table 9:** Hofstede’s cultural dimensions scores of nine countries.

Cultural dimension	Topic intensity								
	RU	BR	DZ	ES	MX	US	FR	KZ	PL
PDI	93	69	80	57	81	40	68	88	68
IDV	39	38	35	51	30	91	71	20	60
MAS	36	49	35	42	69	62	43	50	64
UAI	95	76	70	86	82	46	86	88	93
LTO	81	44	26	48	24	26	63	85	38
IND	20	59	32	44	97	68	48	22	29

RU, Russia; BR, Brazil; DZ, Algeria; ES, Spain; MX, Mexico; US, United States; FR, France; KZ, Kazakhstan; PL, Poland.

The cell phone version is greatly affected by PDI, and IND. PDI have significant positive effects on consumer attention on the cell phone version ( $\beta_{3\_1}$ = 0.213, *p* < 0.01). IND has a significant negative effect on the attention of the cell phone version ( $\beta_{3\_6}$ = −0.227, *p* < 0.01). A unit’s increase in PDI increases consumer attention to the cell phone version by 26%. A unit’s increase in IND leads to a 20% decrease in consumer concern about the cell phone version. Table 9 shows that the values of PDI in Kazakhstan and Russia are high, while that of IND is low. The topic intensity values of the cell phone version in these two countries are larger than those of other countries.

The shopping experience is greatly influenced by LTO, MAS, and PDI. LTO and MAS have significant negative effects on consumer concern about the shopping experience ( $\beta_{14\_5}$ = −0.421, *p* < 0.01, $\beta_{14\_3}$ = −0.248, *p* < 0.01). PDI has a significant positive impact on consumer concern about the shopping experience ( $\beta_{14\_1}$ = 0.196, *p* < 0.01). A unit’s increase in LTO and MAS reduces consumer concern about the shopping experience by 34 and 22%, respectively. A unit’s increase in PDI leads to a 22% increase in consumer concern about the shopping experience. Table 9 shows that the value of LTO and MA in Algeria, Brazil, and Spain are low, while the value of PDI is high. The topic intensity values of the shopping experience in these three countries are large.

LTO, IND, and UAI have a greater impact on shipping speed. LTO and IND have significant negative effects on consumer concern about shipping speed ( $\beta_{1\_5}$ = −0.355, *p* < 0.01, $\beta_{1\_6}$ = −0.257, *p* < 0.01). UAI has a significant positive impact on consumer attention to shipping speed ( $\beta_{1\_4}$ = 0.196, *p* < 0.01). A unit’s increase in LTO and IND decreases consumer attention to shipping speed by 30 and 23%, respectively. A unit’s increase in UAI led leads to a 22% increase in consumer concern about shipping speed. The value of LTO and IND in Poland, Algeria, and Spain are low, while the value of UAI is high. The topic intensity values of shipping speed in these three countries are large.

LTO and UAI have a greater impact on customs dispute. LTO has a significant negative effect on customs dispute ( $\beta_{13\_5}$ = −0.292, *p* < 0.01), while UAI has a significant positive impact on customs dispute ( $\beta_{13\_4}$ = 0.230, *p* < 0.01). A unit’s increase in LTO (UAI) leads to a 25% (26%) decrease (increase) in consumer concern about customs dispute. Compared with other countries, Poland has smaller LTO score and larger UAI score (Table 9), and the topic intensity value for customs dispute is large (Table 4).

Trust in sellers is significantly negatively influenced by LTO ( $\beta_{9\_5}$ = −0.346, *p* < 0.01). MAS and LTO have a significant negative effect on cell phone performance ( $\beta_{5\_3}$ = −0.197, *p* < 0.01, $\beta_{5\_5}$ = −0.172, *p* < 0.01). Consumers from societies with a feminine or short-term oriented culture pay more attention to cell phone performance. Logistics package quality, cell phone function, and items as described are less affected by the cultural dimensions.

For each dimension, PDI has a significant positive impact on logistics service, cell phone versions, and shopping experience, while it has a significant negative effect on customs efficiency and tariff, and value for money. MAS has a significant positive impact on logistics service, while it has a significant negative effect on customs efficiency and tariff. UAI has a strong significant positive impact on shipping speed and customs disputes. LTO has a strong positive impact on logistics service and a strong negative impact on the shopping experience, trust in the seller, shipping speed, customs dispute, and value for money. IND has a strong positive effect on seller recommendation and customs efficiency & tariff but has a strong negative effect on logistics service and shipping speed.

## 5. Conclusion and discussion

### 5.1. Conclusion

Based on an extensive amount of online consumer reviews on Aliexpress, this study provides important insights into service quality improvement from the perspective of cultural differences. We identified the service quality problems consumers are concerned about in CBEC based on the LDA model and analyzed the effect of Hofstede’s cultural dimensions on consumer emotional tendencies and their perception of service quality in the context of CBEC. The main findings are detailed below.

First, consumers pay more attention to service quality in the logistics service, customs efficiency and tariff, shipping speed, shopping experience, cell phone function, cell phone version, and seller recommendation. The main issues that consumers are concerned about in CBEC are logistics service, customs efficiency, tariff, shipping speed, and shopping experience, which is consistent with the previous research on service quality in CBEC (Agatz et al., 2008; Zhao, 2020). Moreover, differences exist among consumers from various cultures regarding their perception of cross-border e-shopping service quality.

Second, cultural traits significantly influence consumers’ emotional tendencies. PDI and IND have significant positive effect on consumer sentiment orientation. On the contrary, UAI has significant negative effect on consumer sentiment orientation. In the negative panel, IDV shows a significant negative effect on consumer sentiment orientation. However, in the neutral panel, IDV shows a significant positive effect on consumer sentiment orientation. In the negative panel, MAS and LTO have a significantly positive effect on consumer sentiment orientation, but in the neutral panel, MAS and LTO negatively affect consumer sentiment orientation. The result is in line with previous studies emphasizing that cultural traits can affect overall service quality expectations (Donthu and Yoo, 1998; Stauss and Mang, 1999; Crotts and Erdmann, 2000; Liu et al., 2001; Reimann et al., 2008; Fang et al., 2013; Huang and Crotts, 2019).

Third, cultural traits affect consumer service quality perception in cross-border e-shopping. PDI, MAS, and LTO have a significant positive impact on logistics service. PDI and MAS have a significant negative effect on customs efficiency and tariff. Consumers with a high degree of IND pay more attention to customs efficiency and tariff. However, their focus on logistics service and shipping speed is relatively low. Although there is no research on the impact of cultural traits on consumers’ service quality perception in the context of CBEC, Sinkovics et al. (2007) proved that consumers who come from a different cultures may have different website service quality perceptions. Cultural traits can effect consumers’ service quality perception in cross-border e-shopping (Donthu and Yoo, 1998).

### 5.2. Theoretical implications

This study provides further evidence to the literature on improvements to e-commerce service quality based on cultural traits and provides a new perspective for CBEC consumer behavior studies on quality improvement.

First, we applied Hofstede’s framework to analyze the influence of cultural dimensions on sentiment orientation and service quality perception in cross-border e-shopping and provides a theoretical basis for the improvement of cross-border e-shopping service quality from the perspective of cultural differences. Our analysis showcases that cultural dimensions not only affect consumer satisfaction but also affect service quality perception. The results suggest that cultural values can influence consumers’ online evaluations and judgments. This is consistent with the research results of Cleveland et al. (2016) who declared that culture can influence customers’ perceptions, filter the income information, and finally make their assessment. Culture influences consumer online behaviors such as service quality exceptions and perceptions (Steinwachs, 1999; Mariani and Predvoditeleva, 2019). Because service is the power that enterprises provide for consumers (Donthu and Yoo, 1998), it is especially important to focus on improving customer service quality from the standpoint of cultural differences at the level of enterprise organization and management (Hofstede et al., 2010; Gnanlet and Yayla-Kullu, 2014).

Second, different from previous research which analyzed some of Hofstede’s dimensions of consumer behavior (Reimann et al., 2008; Mariani and Predvoditeleva, 2019), our research is the first step toward investigating all six of Hofstede’s cultural dimensions of consumers’ online service expectations and perceptions in CBEC, which provide theoretical insights into consumer behavior in CBEC. The results proved that when compared to traditional commerce (Donthu and Yoo, 1998; Hofstede et al., 2010), consumers’ evaluations of CBEC service and perceptions of service quality are quite similar. The findings are also consistent with previous research on the impact of culture on consumer online behavior (Koh et al., 2010; Kwok and Xie, 2016; Wang W. et al., 2018; Huang and Crotts, 2019; Mariani and Predvoditeleva, 2019; Filieri and Mariani, 2021). This implied that culture has an equal impact on consumer service evaluation and perception in traditional commerce and CBEC.

### 5.3. Practical implications

Targeted advertising contributes to increased corporate profits (Jiang and Wu, 2022). Analyzing customer behavior and developing targeted marketing activities can improve service quality and, as a result, customer satisfaction (Wang et al., 2022). Providing differentiated services to customers based on cultural traits improves cross-border e-commerce enterprise lean management and increases corporate profits. As we can see from the results, cultural dimensions have significant impacts on consumers’ emotional tendencies. From the angle of application, sellers should improve service quality for customers from countries characterized by low power distance, masculinity, and restraint and pay significant attention to the main quality problems consumers are concerned about. Truthful descriptions of products should be provided to avoid the negative emotions caused by inconsistencies between the physical goods and product descriptions (e.g., the battery’s standby time) (Mou et al., 2020). Live-streaming e-commerce has significant advantages in visibility, interactivity, real-time, and entertainment, thus reducing product uncertainty and enhancing consumers’ purchase intention (Guo et al., 2022). Sellers can use live vidoes to reduce customer dissatisfaction caused by commodity uncertainty. A few attributes, such as price, have a negative impact on consumers’ positive emotions. Therefore, sellers should explore reasonable pricing mechanisms and improve service quality for high-priced mobile phones and the corresponding supporting services.

The results showed that the perception of service quality is influenced by national culture. Sellers can improve their personalized consumer service and marketing strategies according to consumers’ cultures. They can provide high-quality pre-, in-, and after-sale services to customers from countries characterized by short-term orientation, femininity, and high power distance, and maintain active communication with them through emails or in-site letters regarding service problems related to logistics delivery, consultation, customs clearance, tariff, and return process. They should also improve the shipping speed for consumers from countries characterized by short-term orientation, self-restraint, and strong uncertainty avoidance. Furthermore, since customer perceptions regarding logistics packaging quality, cell phone functions, items as described, and gifts are less affected by cultural differences, sellers can improve the quality of the aforementioned aspects without considering consumers’ cultural traits.

### 5.4. Limitations and future research

This research suffers from several limitations. First, our research objects are cell phones. Considering the particularity of CBEC logistics transportation, the volume and weight of different kinds of goods may affect the choice of mode of transport, which will lead to different shopping experiences for consumers. Therefore, further research on this topic requires in-depth analysis, especially on commodity attributes (Peterson et al., 1997; Lian and Lin, 2008), logistics, and transportation modes. Second, limited by data availability, we only analyzed the impact of cultural differences on consumers’ cross-border e-shopping behavior based on online consumer review data and the location (country) of consumers. Future research should also examine how personal information (e.g., occupation and income) affects consumer behavior. More detailed personalized marketing and customer service strategies are required as a reference for improving service quality. Third, against a backdrop of multi-polarization, economic globalization, and cultural diversity, the cultural traits of countries will also change. How to analyze the impact of cultural differences on CBEC consumer behavior based on the latest national cultural traits is another problem that requires further analysis.

## Data availability statement

The raw data supporting the conclusions of this article will be made available by the authors, without undue reservation.

## Author contributions

LH: conceptualization, validation, data curation, writing—review and editing, and funding acquisition. LH and XH: methodology and writing—original draft preparation. All authors have read and agreed to the published version of the manuscript.

## Funding

This research was funded by the Science of Education Research Project of Shanghai Municipal Education Commission (grant no. C19055).

## Conflict of interest

The authors declare that the research was conducted in the absence of any commercial or financial relationships that could be construed as a potential conflict of interest.

## Publisher’s note

All claims expressed in this article are solely those of the authors and do not necessarily represent those of their affiliated organizations, or those of the publisher, the editors and the reviewers. Any product that may be evaluated in this article, or claim that may be made by its manufacturer, is not guaranteed or endorsed by the publisher.

## Footnotes

^1^https://www.hofstede-insights.com/
